# Supplementary material for: Role of Thalamic CaV3.1 T-Channels in Fear Conditioning
Source: Int J Mol Sci. 2025 Apr 9;26(8):3543. doi: 10.3390/ijms26083543 (PMC12026627; doi:10.3390/ijms26083543)
Supplement: Supplementary file 1 [file ijms-26-03543-s001.zip › ijms-3516011-supplementary.pdf]

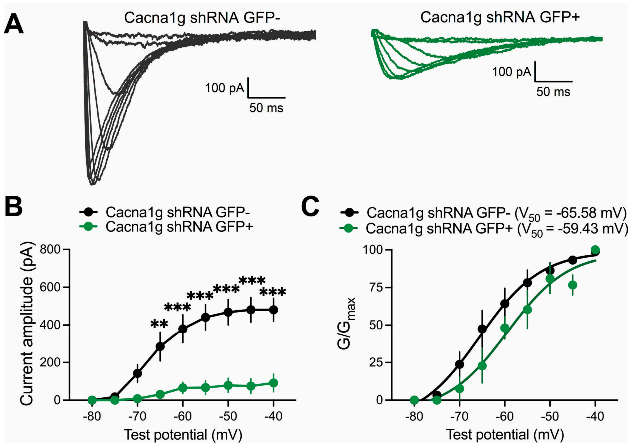

Figure S1. Biophysical properties of CMT T-currents in Cacna1g shRNA GFP-positive and GFP-negative neurons. (A) Representative T-current traces from thalamic GFP-negative (black) and GFP-positive (green) Cacna1g shRNA neurons in voltage range for test potentials of −80 to −40 mV from an initial holding potential of −90 mV in 5 mV increments. Note that half of the GFP-positive neurons did not have T-currents. T-currents recorded from GFP-positive neurons were smaller and did not show typical crisscrossing pattern (green trace). **(B)** Average T-current amplitude, as calculated from steady-state activation protocol, was reduced in Cacna1g shRNA GFP-positive neurons in comparison to GFP-negative cells (two-way RM ANOVA: interaction $F_{(8,96)} = 12.46$, $p < 0.001$; test potential $F_{(8,96)} = 25.75$, $p < 0.001$; GFP $F_{(1,12)} = 26.52$, $p < 0.001$; Sidak's post hoc test). N=7 cells per group; note that 3 GFP-positive cells did not have T-currents. *p<0.05, **p<0.01, ***p<0.001. **(C)** Difference between V_{50} of steady-state activation (G/G_{max}) for GFP-positive and GFP-negative CMT cells was not statistically significant (p>0.05).
